# Miscellaneous Intelligence

**Published:** 1800-03

**Authors:** 


### *Miſcellaneous Intelligence.*

The government of Sweden has probably had more reaſon than any other, to fix its attention on the uſe of coffee. This beverage is now almoſt generally uſed in that country, and will probably become ſtill more general, while it is expenſive in Sweden, as that country is one of the remoteſt countries from the climates where it is produced, and poſſeſſes no colonies which could furniſh her with that article.

Several years ſince, a decree was proclaimed, forbidding the uſe of this favourite decoction; a law which, as might be expected, did not long maintain its effect, ſo that a renewal of it became neceſſary. In the mean time, a Mr. Cavander publiſhed an account of a ſubſtitute for coffee, compoſed of the flower of rye, and yellow Engliſh potatoes, which are much ſweeter than any other ſort. Theſe ingredients are firſt boiled, then made into a kind of cake, which is dried in an oven, and afterwards reduced to a powder reſembling ground coffee. The inventor has procured a certificate from the Royal Medical College at Stockholm, ſtating, that this beverage verage is very ſimilar to coffee in its taſte, as well as in other properties, and that its compoſition is not in the leaſt detrimental to health.

From a number of experiments made on this ſubject, in ſeveral countries, and almoſt every where without ſucceſs, it is matter of regret that we are obliged to doubt the good reception of this factitious coffee in Sweden; though ſeveral merchants who deal in it, have lately publiſhed their addreſſes to the public. It would perhaps be leſs difficult to make the Swedes abandon the uſe of wine, than of *real* coffee. In the cold climates, the inhabitants may eaſily ſupply the want of wine, by ſtrong beer, and other liquors; while coffee, which is more requiſite than in hot climates, cannot be effectually replaced by any other ſubſtitute. *Le Nord Litteraire*, No. 8. p. 366.

---

Dr. G. H. Piepenbring, a phyſician in Hanover, has lately publiſhed a pamphlet, in which he eſpecially recommends the well known root of ſcarcity, or *beta cicla*, and ſeveral other ſpecies of the beta, as excellent *ſubſtitutes for coffee*; in the ſame treatiſe he propoſes the leaves of the raſpberry, or the *rubus idæus*, L. inſtead of the more expenſive and leſs wholeſome tea-leaves; and gives particular directions for preparing both his native tea as well as coffee. This attempt at ſupplanting exotic drugs by indigenious productions, is the more laudable, as the Germans are not leſs addicted to the immoderate uſe of coffee than the Swedes, while their late intercourſe with the Engliſh has likewiſe introduced among them the daily and habitual uſe of tea, which was formerly drank only as a diluent in febrile and catarrhal indiſpoſitions.

---

Another German naturaliſt, Mr. Mitsching, has inſerted a circumſtantial account of a new ſubſtitute for coffee in the Literary Gazette of Dreſden, No. 35, for the year 1797. He praiſes the fruit of the wild dog-roſe, or *roſa arvenſis*, L. as a valuable ſubſtitute, or at leaſt as an addition to coffee, which imparts to it a fine flavour and colour. This berry may, according to him, be preſerved for a whole year in a ſtate proper for roaſting it like coffee, if ſplit in two equal parts, and kept in a dry and temperate place. To determine the length of time required for roaſting this native coffee, as well as to aſcertain the colour it ought to aſſume, the inventor ingenuouſly directs the œconomiſt to make the firſt experiment with equal quantities of foreign coffee, and the berries before ſuggeſted; as ſoon as the coffee has acquired a proper colour, the berries will then likewiſe be ſufficiently roaſted. From his experience, two-thirds of the latter, with one-third of the former, produce excellent coffee; but, when friends are invited vited to partake of it, he advises to invert the proportion of the ingredients.

---

In one of the lateft Numbers of the "*Journal de Phyfique*," we met with an ingenious paper by Dr. BUNIVA, Prof. of Medicine at the Univerfity of Turin, containing an account of a variety of experiments tending to elucidate the doctrine of animal life, of the fecretions, excretions, and effufions, natural as well as morbid; while the author endeavours more efpecially to prove that the red part of the blood, in a living animal, is retained in its proper refervoirs by the force of the vital activity of the parts, rather than by any defect in the capacity of the veffels and pores through the volume of globules. Some of thefe curious experiments we propofe to communicate to the reader, in a future Number.

---

From the fame Journal, we learn that the *fiuat of alumine* has been fent from Copenhagen to the Mineralogical School of Paris. This mineral is a whitifh ftone, femi tranfparent, lamellated, and tolerably foft; it is compofed of the fluoric acid and alumine.

---

The Society of Natural Hiftory at Paris, being anxious to refume their labours, have lately publifhed a new and interefting volume of their Memoirs, in quarto, price fix francs. The manufcripts for feveral other volumes are in hand, and will forthwith be regularly publifhed, if the prefent volume fhould receive the fanction of the public. The editors of the Journal de Phyfique obferve, on this occafion, that there cannot be the leaft doubt of the favourable reception of this work, as it contains memoirs written by the following authors: viz. Lamarck, Haüy, Cuvier, Ventenat, Geoffroy, Latreille, Decandoles, Gillet Laumont, and Leliévre.

---

From the fplendid and valuable "Travels in the Southern Provinces of the Ruffian Empire," by P. S. PALLAS, lately publifhed in Germany, and of which an Englifh tranflation is confiderably advanced in the prefs, we fhall extract the following ufeful hints: The author informs us, that M. Subof, the prefent director of economical affairs in the government of Penfa, has publifhed a treatife in the Ruffian language, pointing out the means of increafing the quantity of fpirits in diftiliation. From a tchetvert of corn or rye, weighing 360 Ruffian pounds, he has, with the ufual apparatus, produced fix eimers and a quarter of fpirits; or, by computing the Ruf. eimer at thirteen quarts, upwards of twenty gallons Englifh liſh meaſure; while others, from a ſimilar quantity of grain, could diſtil only five eimers, or about ſixteen Engliſh gallons and one-fourth: nay, he aſſures the public, that he has brought the art of diſtilling to ſuch perfection, that from 400 Ruſſian pounds weight of grain, he has uniformly obtained ſeven and five-eights eimers of proof-ſpirits. This remarkable increaſe of the produce, the author attributes principally to the following circumſtance. In order to reduce the temperature of the hot water uſed in the maſh, he cauſed cold water and ice to be added, by which the loſs of ſpirituous particles, during the fermentation, was prevented.

---

Another remarkable fact, in the department of Natural Hiſtory, but particularly applicable as a counterpart to the modern rage for *coſmetics*, we ſhall communicate to our readers, in the author's words, from the firſt volume of his Travels, in German, p. 232 and following:

"On the 20th of Auguſt," ſays M. Pallas, "the commandant of the Port of Aſtrakhan, granted me a barge for an excurſion to the mouth of the Volga, a diſtance from ſixty to eighty verſts. As I had learned from the late Dr. Lerch, that the *Nymphæa Nelumbo* grew there in abundance, I ſent a party of my eſcort, who procured a great number of the flowers and fruits of this plant, in different gradations of growth; ſome of which had nearly attained perfect maturity. Theſe fruits are by the Ruſſians called ſea-nuts, or *morſkye orechi*; by the inhabitants of Tybet, *badma*; by the Perſians, *dariopacta*; and by the Indians, *pabin* or *lilifar*; they are ſearched for and eaten with avidity by the laſt-mentioned nation, who regard them as ſacred. According to their Mythology and that of Tybet, the perfect divinities are regenerated in the richly ſcented flowers of this plant, which ſerve them for a throne. Theſe flowers, indeed, have an agreeable flavour; and the diſtilled water which the apothecary of this city, Aſſeſſor Zettler, had the politeneſs to prepare for me, contained a pleaſant and permanent taſte of fine ambra, and when uſed as a lotion, imparts ſuch a ſoftneſs and delicacy to the ſkin of the face and hands, that it deſerves to be introduced as an *innocent cosmetic* into all the apothecaries ſhops; eſpecially as the flowers may be collected throughout the ſummer, in the inlets of the Volga and the Bolda. I found the leaves of this plant completely free from zoophytes and other aquatic inſects. The ſeminal veſſels are more conſpicuous, and the germ more magnified, in the ſeed of the *Nymphæa Nelumbo*, than in any other plant. When the nuts are incloſed in a lump of clay, and immerſed under water, they readily germinate, particularly if a ſlight inciſion be previouſly made in the ſhell."

---

The German Reviewer of Dr. Beddoes's late Treatise on Consumptions, in the Jena Universal Literary Journal, expresses great surprize that the author has made no mention of the propagation of the disease by contagion; and thinks, that Dr. Beddoes's own observation of whole families having been exterminated by it, should have led him to enlarge upon that topic. The opinion of the contagious nature of phthisis, is still universally prevalent among the German physicians, who seem not yet to have learnt, that its being so peculiarly fatal to particular families, depends upon a certain hereditary conformation, or predisposition of the body, not upon its being propagated by contagion from one member of the family to the other.

---

Dr. Scherf, Aulic Counsellor at Detmold, continues his "Beyträge zum Archiv der Medicinischen Policey," (Contributions to the Archives of Medical Police), which ended with the eighth vol. upon an improved plan, under the title of "Isis, oder Journal der Ærztlichen, Gesetzgebung und der oeffentlichen Gesundheitspflege," (Isis, or Journal of Medical Jurisprudence and Police.)

---

